# Book Review: “Cerebral Small Vessel Disease”. What’s the Big Deal about Small Vessels?

**DOI:** 10.3389/fneur.2015.00175

**Published:** 2015-08-10

**Authors:** Andreas Charidimou

**Affiliations:** ^1^Department of Neurology, Massachusetts General Hospital Stroke Research Center, Harvard Medical School, Boston, MA, USA; ^2^UCL Institute of Neurology and The National Hospital for Neurology and Neurosurgery, London, UK

**Keywords:** stroke, cerebral small vessel disease, intracerebral hemorrhage, MRI, biomarkers, cerebral amyloid angiopathy, white matter hyperintensities, lacunes

Cerebral small vessel disease is perhaps among the most common pathologies in the aging brain, primarily affecting the small perforating arteries and arterioles in the cortex and underlying structures of the white and deep gray matter. Small vessel disease includes hypertensive arteriopathy (arteriolosclerosis, fibrohyalinosis, or lipohyalinosis) and cerebral amyloid angiopathy (injury to the vascular wall caused by deposition of the amyloid-β) as well as a range of less common genetic/hereditary or other forms with various etiologies (1). These small vessel disease processes can lead to vessel occlusion with small subcortical (lacunar) infarcts – accounting for a third of symptomatic strokes, or to vessel rupture with spontaneous intracerebral hemorrhage. Intracerebral hemorrhage, in particular, is the most severe and lethal type of stroke. The clinical importance of small vessel disease goes beyond causing obvious acute stroke syndromes, since it is also the commonest cause of “silent” strokes with cumulative effects on cognition. In fact, asymptomatic small vessel disease revealed by MRI, such as leukoaraiosis, cerebral microbleeds, etc., play a key role in vascular cognitive impairment and dementia, one of the biggest challenges facing all aging societies.

The pathophysiological and clinical spectrum of small vessel disease continues to expand rapidly, as revealed by the increasing number of published reports each year. Despite being known to pathologists for decades, as the resolution of brain MR imaging grows, parenchymal injury associated with small vessel disease is unraveled *in vivo*, creating many clinical dilemmas in stroke medicine, dementia, and aging. Hence, small vessel disease is of key interest to a broad scientific and clinical community.

*Cerebral small vessel disease* (Figure 1) is a 371-page multi-authored textbook attempting to bring all sources of information together in a single volume, to summarize the entire current knowledge, and controversies in the field. The book covers a range of topics, from pathological, pathogenic, and genetic aspects, to current neuroimaging (clinical and research) methods, biomarkers, and various clinical aspects. The volume is organized in four sections. The first covers basic definitions, classification, pathology, and basic aspects. Three chapters devoted in key ischemic and hemorrhagic consequences of small vessel disease, achieve to reaffirm the geography of pathology, and bring the subject to life. The second section deals with various neuroimaging and laboratory aspects, both in the routine clinical setting and new approaches to image small vessel disease. The third section discusses the clinical consequences of small vessel disease in specific settings and its role in cognition and disability. Finally, the last section poses an interesting approach in the treatment of small vessel disease, with new hypotheses on trial design and a glimpse into the future of the field.

**Figure 1:**
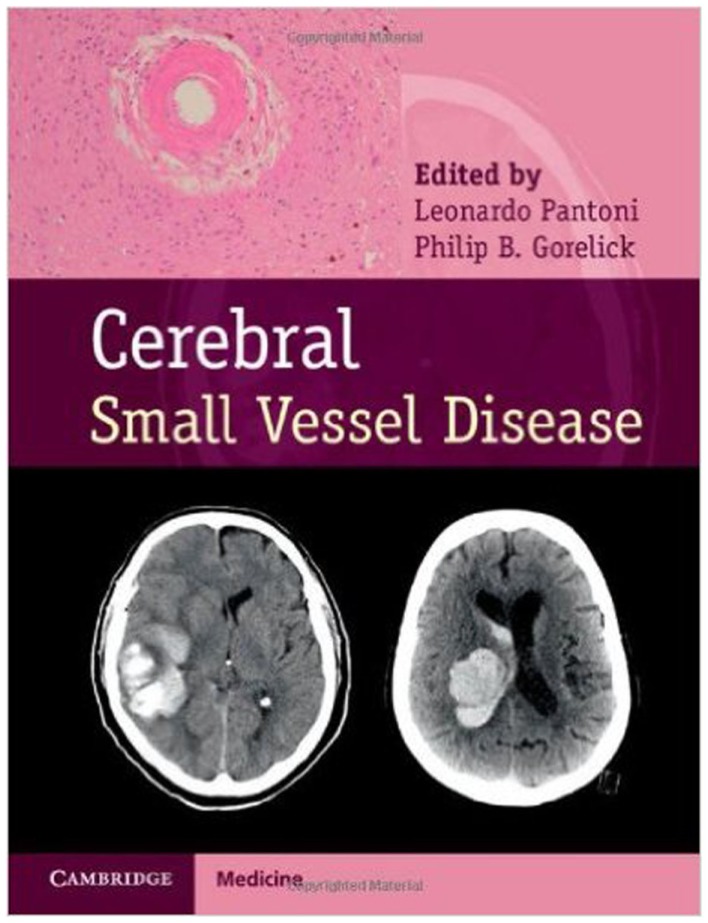
**Cerebral Small Vessel Disease, by Leonardo Pantoni and Philip B. Gorelick (Eds.)**. Reprinted with permission from Cambridge University Press.

One of the few aspects not systematically covered in the book is an informative discussion on MRI-visible perivascular spaces, a relatively new marker of small vessel disease and a current hot topic in research. In addition, the chapters dealing with hemorrhagic aspects of small vessel disease are somewhat under-represented in this volume. Chapter 4 focusses on the pathologic consequences of hemorrhagic small vessel disease, providing an excellent overview of the topic. However, a chapter dedicated to all the different aspects of spontaneous intracerebral hemorrhage is missing; instead, this important topic rather appears patchy within different chapters. I also feel that the clinical and pathophysiological spectrum of cerebral amyloid angiopathy is not captured in its entirety. Chapter 13 “Imaging of hemorrhagic cerebral small vessel diseases” partly compensates for this by presenting an excellent modern summary of the key imaging findings and clinical types of amyloid angiopathy, including cerebral microbleeds and cortical superficial siderosis, but some of these aspects might have benefited by more detailed individual chapters going in greater depth. Similarly, the possible contribution of cerebral amyloid angiopathy in cognitive impairment and dementia is only briefly covered. Finally, clinicians should not expect a book with evidence-based recommendations on how to manage their patients with all different MRI lesions suggestive of small vessel injury, partly since there are, as yet, no reliable data to inform clinical guidelines on specific treatment settings, such as thrombolysis, oral anticoagulation, etc. Despite this, the authors cover in a comprehensive way the current evidence where available.

Overall, the Editors have done a remarkable job in assembling a world-class team of authors, both established authorities in their fields as well as rising stars. As is often the case with books of this kind, given the relatively long time required to collect a multi-author book, several important new findings made in the interim might be missing. Due to the diverse co-author teams, the style varies between chapters, but the reader will benefit from all the different perspectives. Although each chapter can be read as a stand-alone piece, the book flows nicely with repetition between chapters minimized as much as possible – it can hence also be used as a single comprehensive volume. This book will be of interest to all clinicians and researchers working in the fields of stroke and cognitive impairment, including students or trainees new to the field, wanting to know what is the big deal about small vessels as well as the more experienced clinician or neuroscientists in the field.

The book goes a long way synthesizing practical clinical knowledge and basic aspects in small vessel disease and in bridging the gap between what we see on MRI, what we know, and what we can do, on this fascinating topic. Definitive answers to many questions cannot yet be provided, but this volume will contribute significantly in understanding all that is known and directions for future research. The editors and authors have rendered a great service by putting this piece together. To quote Steven Greenberg, “There is nothing small about the consequences of small-vessel disease” (2), and arguably cerebrovascular diseases and cognitive impairment are becoming the leading cause of death and disability worldwide. The publication of this book is timely as it coincides with major advances and a great momentum in the field (3).

## Conflict of Interest Statement

The author declares that the research was conducted in the absence of any commercial or financial relationships that could be construed as a potential conflict of interest.
